# Distinct T Cell Receptor Clonotypes in the Sardinian Population Highlight the Role of Mucosal‐Associated Invariant T Cells and Invariant Natural Killer T Cells in Multiple Sclerosis

**DOI:** 10.1111/imm.70013

**Published:** 2025-06-29

**Authors:** Jiaxin Shen, Elena Rita Simula, Yisu Liu, Gustavo Sganzerla Martinez, Xiaofen Wen, David J. Kelvin, Leonardo A. Sechi

**Affiliations:** ^1^ Department of Hematology The First Affiliated Hospital of Shantou University Medical College Shantou Guangdong People's Republic of China; ^2^ Department of Biomedical Sciences University of Sassari Sassari Italy; ^3^ Division of Immunology International Institute of Infection and Immunity, Shantou University Medical College Shantou Guangdong China; ^4^ Department of Microbiology and Immunology & Canadian Centre for Vaccinology Dalhouise University Halfax Nova Scotia Canada; ^5^ Department of Medical Oncology Cancer Hospital of Shantou University Medical College Shantou Guangdong People's Republic of China; ^6^ SC Microbiologia e Virologia Azienda Ospedaliera Universitaria Sassari Italy

**Keywords:** MAIT and iNKT cells, multiple sclerosis (MS), Sardinian population, T cell receptor (TCR) clonotypes

## Abstract

Multiple sclerosis (MS) is a chronic, autoimmune, inflammatory disease of the central nervous system, driven by T‐cell mediated immune responses. Studying T‐cell receptor (TCR) clonotypes specific to MS in distinct populations can provide insights into disease mechanisms. The Sardinian population, with its unique genetic background resulting from geographical isolation, presents a high‐risk cohort for MS research, offering a valuable context for understanding the disease's pathogenesis. We analysed the frequency of unique TCR clonotypes in peripheral blood samples from Sardinian MS patients and healthy controls, focusing on TCRα and TCRβ CDR3 sequences. Clonotypes were functionally annotated for antigen‐specific interactions, and hierarchical analysis was performed to identify shared TCR clonotypes between MS patients and healthy controls. A total of 119 TCRβ and 521 TCRα CDR3 clonotypes were significantly more frequent in MS patients compared to healthy controls (*p* < 0.05). Several TCR‐α clonotypes, such as CAVLDSNYQLIW (a MAIT cell clonotype targeting the BST2 antigen) and CAVNTGNQFYF (cross‐reactive to multiple antigens, including CMV p65 and BST2), were identified as specific to MS. Shared clonotype analysis revealed the involvement of mucosal‐associated invariant T (MAIT) cells and invariant natural killer T (iNKT) cells in MS pathogenesis. Notably, HCV‐specific TCR‐α clonotypes (NS3‐HCV) were significantly increased in MS patients, suggesting a link between infectious disease‐related and autoimmune‐related clonotypes. No significant differences were observed for other antigens, such as VP22, p65 and BST2. This study identifies distinct TCR clonotypes associated with MS in the Sardinian population, highlighting the role of MAIT and iNKT cells in the disease's pathogenesis. The findings suggest that HCV‐specific TCR repertoires may contribute to the development of MS. These results improve our understanding of T‐cell mediated immune mechanisms in MS and offer potential targets for therapeutic intervention, particularly within the Sardinian cohort.

## Graphical abstract

Antigen‐specific TCR‐α clonotype frequencies in MS and HC groups. (A) Heatmap of CDR3 TCR‐α clonotype annotations. (B) Comparative analysis of CDR3 TCR‐α clonotypes targeting viral and autoimmune‐related antigens, including VP22, p65, NS4B, MLANA, M1, HA and BST2. NS3‐HCV‐specific CDR3 clonotypes are significantly enriched in MS patients.

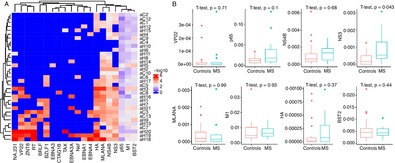

## 1 Introduction

Multiple sclerosis (MS) is a chronic, inflammatory and demyelinating disease of the central nervous system (CNS) characterised by autoimmune attacks against myelin sheaths, leading to neurological dysfunction [1]. T‐cell mediated immunity plays a pivotal role in MS pathogenesis [2], with autoimmune T‐cell responses to myelin proteins, including myelin basic protein (MBP) [3], myelin oligodendrocyte glycoprotein (MOG) [4] and proteolipid protein (PLP) [5], being central to disease progression. These T‐cells are present in MS brain lesions and peripheral blood, suggesting that they contribute to disease pathology in both experimental models and human MS [6, 7]. Despite the well‐established role of T‐cells, the aetiology of MS remains unclear.

Recent studies have highlighted the potential role of environmental factors, particularly viral infections, in triggering or exacerbating MS. Pathogens such as Epstein–Barr virus (EBV) [8], *Chlamydia pneumoniae* [9] and retroviruses [10] have been implicated, though the exact mechanisms remain debated. Elevated EBV serological levels in MS patients compared to healthy individuals suggest a potential link, with one hypothesis proposing that EBV infection triggers MS through molecular mimicry, where EBV antigens resemble CNS self‐antigens, initiating an autoimmune response [11, 12]. This underscores the possibility that viral infections interact with genetic factors to initiate or worsen MS.

In addition to environmental triggers, genetic factors, particularly human leukocyte antigen (HLA) genotypes, are well‐established risk factors for MS. The HLA alleles include DRB1*15:01, DRB1*03:01 and DRB1*13:03, which are strongly associated with MS susceptibility in European populations [13, 14, 15, 16]. Sardinia, with its genetically isolated population and high MS prevalence, offers a unique opportunity to study MS genetics and immunology [13, 17, 18]. Investigating MS‐specific TCR clonotypes in this cohort could provide valuable insights into the immune mechanisms underlying MS, potentially identifying biomarkers for early diagnosis and therapeutic targets.

T‐cell receptor (TCR) cross‐reactivity is proposed as a key mechanism in autoimmune diseases, where TCRs with broad specificity may recognise a range of antigens [19]. In MS, cross‐reactive TCRs may target self‐antigens, leading to CNS autoimmune attacks. This study aims to characterise MS‐specific TCR clonotypes in the Sardinian population by focusing on the analysis of TCRα and TCRβ CDR3 sequences. By examining the frequency of these clonotypes in MS patients and healthy controls, we seek to uncover novel immunological signatures associated with the disease. Additionally, we explore the potential link between infectious disease‐related and autoimmune‐related TCRs, particularly focusing on clonotypes related to viruses such as hepatitis C virus (HCV), which has been suggested to have a role in MS pathogenesis. Our findings may provide a deeper understanding of the T‐cell mediated immune mechanisms driving MS and could identify new avenues for personalised immunotherapies or early diagnostic biomarkers in MS.

## 2 Materials and Methods

### 2.1 Participants and Ethics

This study was approved by the ethics committee of the University of Sassari, Sardinia, Italy. MS patients (as listed in Table 1) were enrolled at the University of Sassari according to the McDonald criteria [20], and healthy volunteers were enrolled from the Immunohematology and Transfusion medicine of the University of Sassari enrolled in this study after informed consent.

**TABLE 1:** Basic information of multiple sclerosis patients.

ID	HLA	Gender	Age (years)	Period between initial onset (years)	Relapses
C1	HLA‐DRB1*0301‐DQB1*0201 HLA‐DRB1*0301‐DQB1*0201	F	54	25	2015/12/1
C2	HLA‐DRB1*0301‐DQB1*0201 HLA‐DRB1*0301‐DQB1*0201	F	47	13	NO
C3	HLA‐DRB1*0301‐DQB1*0201 HLA‐DRB1*0301‐DQB1*0201	F	48	16	NO
C6	HLA‐DRB1*0301‐DQB1*0201 HLA‐DRB1*0301‐DQB1*0201	F	43	11	NO
C7	HLA‐DRB1*0301‐DQB1*0201 HLA‐DRB1*0301‐DQB1*0201	F	34	10	NO
C12*	HLA‐DRB1*0301‐DQB1*0201 HLA‐DRB1*0301‐DQB1*0201	F	26	7	NO
C5*	HLA‐DRB1*0301‐DQB1*0201 HLA‐DRB1*0301‐DQB1*0201	M	44	10	NO
C4*	HLA‐DRB1*0405‐DQB1*0301 HLA‐DRB1*0405‐DQB1*0302	F	43	9	NO
C8	HLA‐DRB1*0405‐DQB1*0301 HLA‐DRB1*0405‐DQB1*0301	F	47	22	NO
C9	HLA‐DRB1*0405‐DQB1*0301 HLA‐DRB1*0405‐DQB1*0302	F	62	12	NO
C10	HLA‐DRB1*0405‐DQB1*0301 HLA‐DRB1*0405‐DQB1*0302	M	66	50	2016/4/1
C11	HLA‐DRB1*0405‐DQB1*0301 HLA‐DRB1*0405‐DQB1*0301	F	42	10	2015/11/1
C13*	HLA‐DRB1*0405‐DQB1*0301 HLA‐DRB1*0405‐DQB1*0302	F	47	21	NO

### 2.2 Peripheral Blood Mononuclear Cells Isolation and RNA Extraction

A 4 mL peripheral blood was collected into EDTA tubes. To isolate peripheral blood mononuclear cells (PBMCs) Ficoll‐Paque PLUS (GE Healthcare Health Sciences) was used, density gradient centrifugation was performed, and the cells were washed with phosphate buffered saline (PBS). PBMCs were added into TRIzol (Thermo Fisher Scientific Inc., USA) for RNA extraction. RNA concentration and purity were measured with an Agilent 2100 bioanalyzer (Agilent Technologies, Palo Alto, CA).

### 2.3 Amplification of TCR Library for Next Generation Sequencing

TCR library for next generation sequencing (NGS) was synthesised by using the Human TCR Profiling (Clontech Laboratories Inc., Mountainview, CA, USA) following the manufacturer's instructions. Briefly, the isolated RNA was synthesis first‐strand cDNA by MMLV‐derived SMARTScribe Reverse Transcriptase (RT). Then full‐length cDNAs process amplification by two round nest PCR. The adapter and index fit for the Illumina sequencing platform (read 2 + i7 + P7 and read 1 + i5 + P5, respectively) were induced in the reserve primer of the second round PCR amplification. The final PCR product was purified by Agencourt AMPure XP Reagent beads (Beckman Coulter Inc., CA, USA). Library quality validation was tested by Agilent 2100 bioanalyzer (Agilent Technologies, Palo Alto, CA). To test concentration and size the KAPA Library Quantification Kits (Kapa Biosystems, Wilmington, MA, USA) was used.

### 2.4 Illumina Next Generation Sequencing

The final TCR library was shipped to NGS company BGI China (BGI diagnostic Laboratories, Shenzhen, China) for performance. The library was sequenced on the Illumina MiSeq sequencer using the 600‐cycle MiSeq Reagent Kit v3 (Illumina, Cat. No. MS‐102‐3003) with paired‐end, 2 × 300 base pair reads.

### 2.5 Next Generation Sequencing Data Analysis

Firstly, the initial quality control assay was used FastQC [21]. Adapter and quality trimming as well as pairing Fastq‐join were perform by ea‐utils [22]. The joined fastq rerun the fastQC for the quality control. TCR alignment and αTCR/βTCR clonotypes exported by MIXCR [23] for downstream analysis. VDJtools [24] was applied for the analysis the data post‐MIXCR for comparative post‐analysis of TCR repertoires include basic statistical analysis, diversity estimation and repertoire overlap analysis. TCR antigen specificity annotation analysis by VDJdb [24]. Statistical analysis was performed using GraphPad Prism 6.0 (GraphPad Software, San Diego, CA) and R package ggplot2 [25] in R version 3.5.0 [26] environment. CDR3 phylogenetic analysis was performed by MEGA5 [27] and Simple Phylogeny in EMBL‐EBI [28]. The phylogenetic tree was drawn by ggtree [29]. TCRlogo performs by software weblogo [30]. Heatmap was constructed by ComplexHeatmap [31].

### 2.6 Statistical Analysis

Statistical analysis was analysed using GraphPad Prism 6.0 (GraphPad Software, San Diego, CA) and R. An unpaired two‐tailed *t* test was performed when comparing two conditions with normal distribution, and an unpaired two‐tailed Mann–Whitney *U* test was used when comparing two samples with abnormal distribution. A one‐way ANOVA and post hoc Tukey test were applied when comparing neonates of different ages with adults. Statistical significance is indicated by *p* values: *p < 0.05, **p < 0.01, ***p < 0.001, ****p < 0.0001.

## 3 Results

### 3.1 Detection of Longer TCRα CDR3 Nucleotide Length Distribution in MS Patients From Sardinia

We sequenced the TCR‐α and ‐β repertoires from peripheral blood samples of Sardinian individuals with MS and healthy controls (HC) to assess the specificity and diversity of the TCR repertoire in MS. The MS cohort (*N* = 13) showed an average of 11 507 ± 3612 functional TCR‐β CDR3 sequences, while the HC group (*N* = 21) had 19 604 ± 3534 sequences.

**FIGURE 1:**
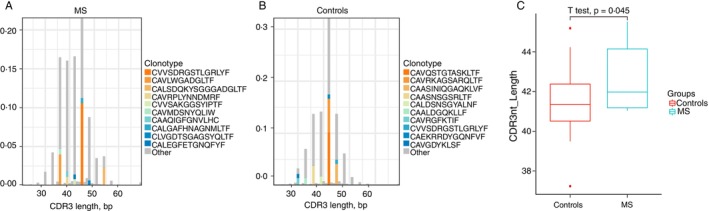
TCR‐α CDR3 nucleotide length distribution in Sardinian MS patients and healthy controls. (A and B) Spectratype plots displaying CDR3 nucleotide (bp) length distributions and the top 10 clonotypes in MS patients (A) and healthy controls (B). (C) Comparative CDR3 nucleotide length distribution of TCR‐α chains between MS patients and HC. Error bars represent SEM.

To reduce PCR amplification bias and ensure accurate analysis, the top 800 clonotypes from the TCR‐α repertoire were selected for comparison. As shown in Figure 1, Spectratype analysis of the top 800 TCR‐α clonotypes from the MS group revealed an abnormal distribution, with the top three clonotypes identified as rearrangements of TRAV9‐2 and TRAJ45, and labelled as CALSDQKYSGGGADGLTF. Analysis of the CDR3 length distribution in the TCR‐α repertoire revealed a significant difference between MS patients (42.73 ± 0.03, *N* = 13) and HCs (41.41 ± 2.62, *N* = 21) (*p* = 0.045).

**FIGURE 2:**
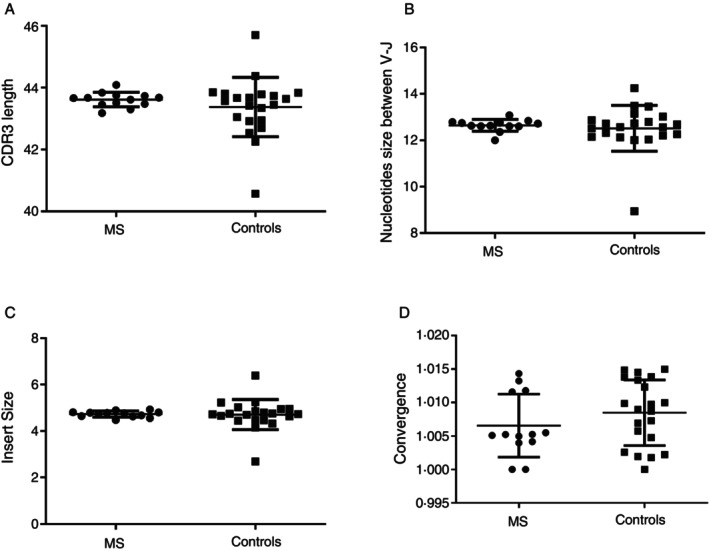
Comparative analysis of TCR‐β CDR3 features between MS patients and healthy controls. (A) Mean CDR3 nucleotide sequence length weighted by clonotype frequency. (B) Number of nucleotides between V and J segments. (C) Mean random nucleotides inserted in CDR3 sequences. (D) Convergence index: Unique CDR3 nucleotide sequences encoding the same amino acid sequence. Data presented as mean ± SEM.

In contrast, no significant differences in CDR3 length were detected in the TCR‐β repertoire (*p* = 0.3837, MS patients 43.62 ± 0.06532, *N* = 13; HCs 43.38 ± 0.2095, *N* = 21) (Figure 2A). Additionally, there was no significant difference in the number of nucleotides between the V and J segments (Figure 2B) or the number of random nucleotides inserted into the CDR3 sequences (MS patients: 4.731 ± 0.03622, *N* = 13; HCs: 4.713 ± 0.1411, *N* = 21) (Figure 2C). The average number of unique CDR3 nucleotide sequences encoding the same CDR3 amino acid sequence was also similar between the MS group (1.007 ± 0.001305, *N* = 13) and the control group (1.008 ± 0.001069, *N* = 21) (*p* = 0.2645) (Figure 2D).

### 3.2 Hierarchical Clustering of TCR Repertoires Reveals Differences Between MS Patients and HCs

**FIGURE 3:**
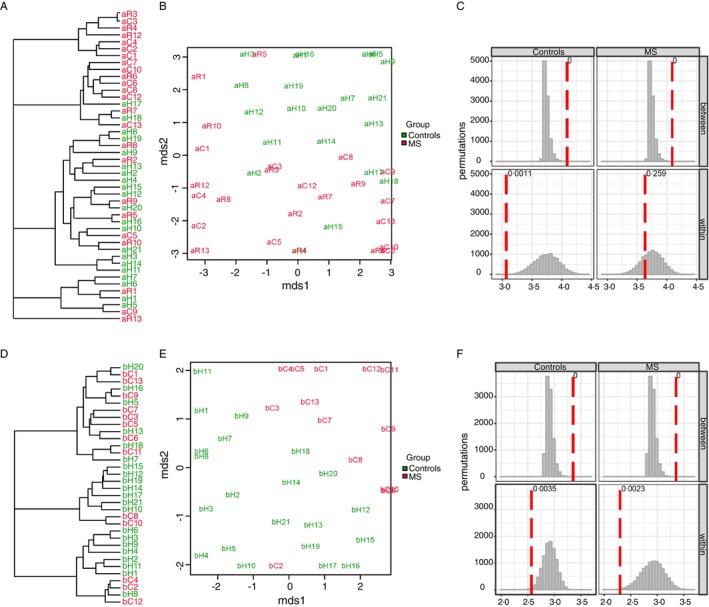
Hierarchical clustering and multidimensional scaling (MDS) of TCR repertoires. (A and D) Dendrograms of TCR α‐chain (A) and β‐chain (D) CDR3 amino acid sequences show distinct separation between MS patients (red) and HC (blue). (B and E) MDS plots confirm spatial segregation of MS (red) and HC (green) groups for α‐chain (B) and β‐chain (E). (C and F) Permutation tests (*n* = 10 000) reveal significant differences between MS and HC groups in α‐chain (C, *p* < 0.05) and β‐chain (F) repertoires.

To further explore differences between MS patients and HCs, we performed hierarchical clustering on the CDR3 amino acid sequences. The resulting dendrograms (Figure 3A,D) demonstrated distinct clustering, clearly separating MS patients from HCs in both the TCR α‐chain and TCR β‐chain repertoires. This separation was further confirmed by multidimensional scaling (MDS) analysis (Figure 3B), where MS patients formed a distinct cluster apart from the control group. A CDR3‐based permutation test (*n* = 10 000) indicated a significant difference between the TCR‐α repertoires of MS patients and HCs (Figure 3C). Analysis of the TCR β‐chain repertoire also revealed notable differences between the two groups (Figure 3E,F).

These findings suggest that specific clonotypes may be driving the observed differences in the TCR repertoires of MS patients. Our next step was to determine what the unique clonotypes of the MS patients are. Furthermore, are these clonotypes responsible for the pathological autoreactive responses of MS?

### 3.3 Biased Usage of Variable (V) and Joining (J) Gene Segments in MS Patients

**FIGURE 4:**
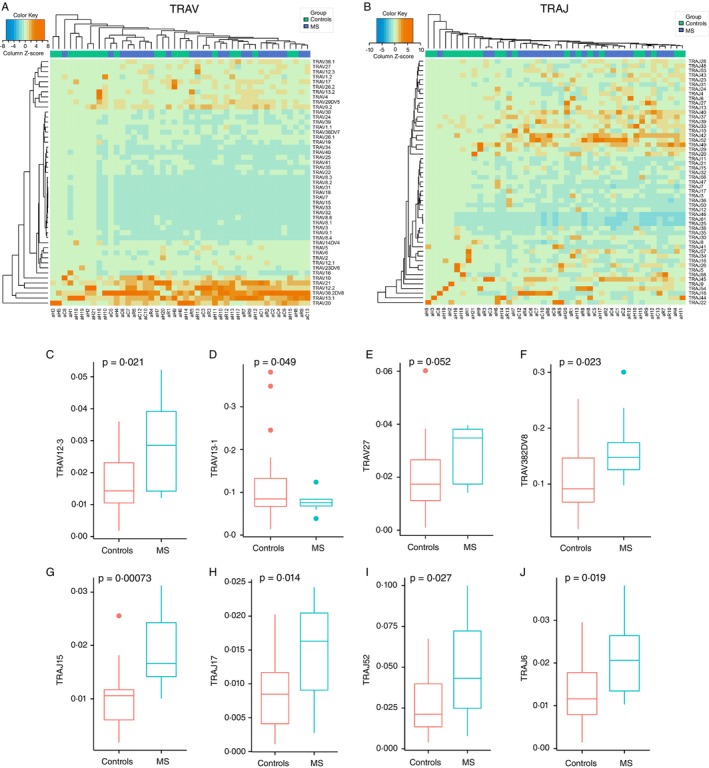
Biased V and J gene segment usage in the TCR‐α repertoire of MS patients. (A and B) Heatmaps of TCRAV (A) and TCRAJ (B) gene usage highlight distinct clustering patterns in MS patients. (C–F) TRAV12.3, TRAV27 and TRAV38‐2DV8 are enriched in MS patients. (G–J) TRAJ15, TRAJ17, TRAJ52 and TRAJ6 show increased frequencies in MS versus HC.

Hierarchical clustering based on Euclidean distance was applied to the V/J gene usage repertoires of MS patients and HCs. While the heatmap of TCRA and TCRB gene usage did not show clear clustering patterns, distinct clustering was observed in specific TCRAV and TCRAJ genes (Figure 4A,B). In TCRAV gene usage, distinct clusters emerged for C7, C2, C1, C9 and C4, while for TCRAJ genes, clustering was noted for C4, C1, C2 and C9. Statistical analysis identified several V and J genes with significantly higher frequencies in MS patients compared to controls. These included TRAV12.3, TRAV27 and TRAV38‐2DV8 (Figure 4C–F), as well as TRAJ15, TRAJ17, TRAJ52 and TRAJ6 (Figure 4G–J). Significant differences in V and J gene usage between MS patients and controls are summarised in Table 2.

**TABLE 2:** V and J gene usage in MS patients and healthy controls.

TCRA	MS	Controls	*p*
TRAV12.3	0.029311	0.0169	0.021307
TRAV13.1	0.075794	0.121643	0.049075
TRAV27	0.029004	0.020036	0.052023
TRAV38‐2DV8	0.159797	0.108271	0.02268
TRAV5	0.012193	0.021722	0.014338
TRAJ11	0.009943	0.006756	0.042563
TRAJ15	0.019014	0.009979	0.000728
TRAJ17	0.014712	0.008432	0.014006
TRAJ52	0.048084	0.024994	0.027462
TRAJ6	0.021035	0.013237	0.018615

**FIGURE 5:**
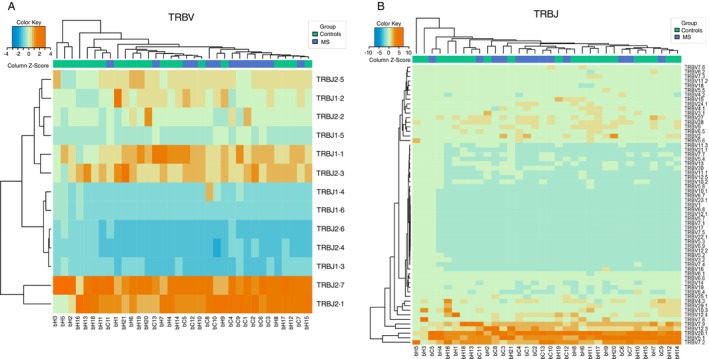
Biased V and J gene segment usage in the TCR‐β repertoire of MS patients. (A and B) Hierarchical clustering of TCRBV (A) and TCRBJ (B) gene usage highlights distinct clustering patterns in MS patients.

No significant clustering or differences were observed in TCR‐β segment usage between the MS group and HCs. However, clustering was observed for C3, C6, C2, C1, C9 and C4 in V gene segments, and for C4, C1, C2, C13 and C10 in J gene segments (Figure 5A,B). Further statistical analysis revealed significantly higher frequencies of TRBV23.1 and TRBV3.1 in HCs compared to MS patients (Table 3). Although TRBV5.6 (*p* = 0.98) and TRBV5.1 (*p* = 0.4811) were previously reported to be associated with MS, no significant differences were found in their usage in this study. Interestingly, TRBV20.1 (*p* = 0.0792) showed higher frequencies in the MS group, which aligns with previous studies in the Sardinian population, though there is insufficient data to confirm its role in increasing MS risk. In the case of TRBJ gene usage, TRBJ1.5 (*p* = 0.004061) and TRBJ1.4 (*p* = 0.03988) were significantly more frequent in MS patients (Table 4).

**TABLE 3:** V gene usage in MS patients and healthy controls.

V gene	Controls	MS	*p*
TRBV23.1	0.000333	0.000101	0.03571
TRBV21.1	0.002154	0.003022	0.05548
TRBV6.4	0.006353	0.003473	0.05544
TRBV5.6	0.019996	0.019841	0.98
TRBV3.1	0.026739	0.016178	0.002162
TRBV5.1	0.098168	0.10764	0.4811
TRBV20.1	0.104435	0.123383	0.0792

**TABLE 4:** J gene usage in MS patients and healthy controls.

J gene	Controls	MS	*p*
TRBJ1.4	0.027317	0.043298	0.03988
TRBJ1.5	0.055716	0.06732	0.004061

Although no significant differences were observed in the usage of TRBV20‐1 between the two groups, we found significant increases in the rearrangement of TRBV20.1$TRBJ2.1 (*p* = 0.01223) and TRBV20.1$TRBJ1.5 (*p* = 0.04875) in MS patients (Table 5). This rearrangement pattern exceeded 1% frequency in the MS group. Analysis of V–J rearrangements confirmed these findings, with TRBV20.1$TRBJ2.1 being the most frequent in the MS‐related CDR3 repertoire, accounting for 5% of all V–J rearrangements. In contrast, the most frequent V–J rearrangement in the control group was TRBV7‐2$TRBJ2.7, at 2.6%.

**TABLE 5:** V–J junction of MS patients and healthy controls.

V–J junction	Controls	MS	*p*
TRBV19$TRBJ1.3	5.3151E‐05	0.00026	0.03939
TRBV21.1$TRBJ2.5	0.00011923	0.00048	0.03879
TRBV5.6$TRBJ1.5	0.00087559	0.00295	0.04661
TRBV5.5$TRBJ1.4	0.0003248913 0.0008485224	0.00085	0.01746
TRBV12.4$TRBJ2.4	0.00024834	0.00061	0.0412
TRBV4.3$TRBJ1.3	0.00041331	0.00096	0.00727
TRBV5.4$TRBJ2.1	0.00034233	0.00072	0.02828
TRBV5.6$TRBJ2.3	0.00102444	0.00211	0.00232
TRBV12.4$TRBJ2.3	0.00154772	0.0029	0.00326
TRBV7.6$TRBJ2.3	0.00139905	0.00253	0.01444
TRBV15$TRBJ1.2	0.00086822	0.00152	0.03137
TRBV4.1$TRBJ1.1	0.00169047	0.00287	0.03532
TRBV12.4$TRBJ1.1	0.00233696	0.00394	0.03131
TRBV4.3$TRBJ1.1	0.00166354	0.00274	0.0108
TRBV19$TRBJ2.7	0.00061349	0.00099	0.0323
TRBV12.4$TRBJ2.2	0.00158877	0.00257	0.02417
TRBV5.6$TRBJ2.1	0.00199412	0.00322	0.00034
TRBV6.6$TRBJ2.5	0.0004826	0.00077	0.01659
TRBV2$TRBJ2.7	0.0025739	0.00377	0.01242
TRBV20.1$TRBJ2.1	0.01718289	0.02225	0.01223
TRBV20.1$TRBJ1.5	0.00808951	0.01031	0.04875

Spectratype analysis of MS‐related CDR3 did not reveal clear biases in the TCR‐β repertoires between MS patients and HCs, although individual V genes exhibited biased usage, possibly due to the limited number of MS‐related CDR3 clonotypes.

### 3.4 Identification of MS‐Specific TCR Clonotypes, Including MAIT and iNK T‐Cells

**FIGURE 6:**
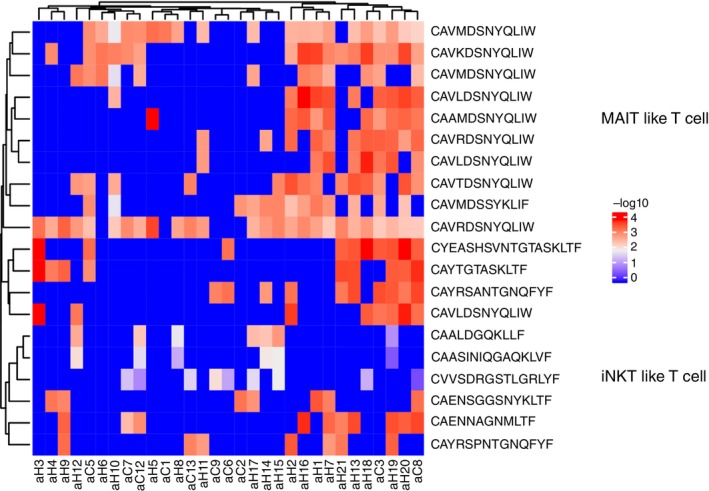
MS‐specific TCR clonotypes associated with MAIT and iNKT cells. Hierarchical analysis of the top 20 clonotypes enriched in MS patients reveals associations with mucosal‐associated invariant T (MAIT) cells and invariant natural killer T (iNKT) cells.

To identify the clonotypes responsible for the observed differences between MS patients and HCs, we analysed the frequency of unique clonotypes, focusing on the top 20. These clonotypes were not only enriched in the MS group but were also functionally annotated for antigen‐specific interactions. Hierarchical analysis revealed that several of these clonotypes were associated with mucosal‐associated invariant T (MAIT) lymphocytes and invariant natural killer T (iNKT) cells (Figure 6). Notably, the TCR‐α CDR3 sequence CAVLDSNYQLIW, a MAIT clonotype, has been shown to target the BST2 (bone marrow stromal antigen 2) antigen. Another clonotype, CAVNTGNQFYF, exhibited cross‐reactivity to multiple antigens, including CMV p65, influenza M1 and BST2 peptide epitopes, while the CAVSNTGNQFYF clonotype was also specific to BST2 (Table 6).

**TABLE 6:** MS associated CDR3 annotation.

CDR3aa (sample)	V (sample)	J (sample)	Epitope gene (DB)	Epitope species (DB)	Reference (DB)
CAVLDSNYQLIW	TRAV1‐2	TRAJ33	BST2	*Homo sapiens*	https://github.com/antigenomics/vdjdb‐db/issues/193
CAVNTGNQFYF	TRAV12‐2	TRAJ49	p65	CMV	PMID:28423320 [32]
CAVNTGNQFYF	TRAV12‐2	TRAJ49	M1	Influenza A	PMID:28636589 [33]
CAVNTGNQFYF	TRAV12‐2	TRAJ49	BST2	*Homo sapiens*	https://github.com/antigenomics/vdjdb‐db/issues/193
CAVSNTGNQFYF	TRAV12‐2	TRAJ49	BST2	*Homo sapiens*	https://github.com/antigenomics/vdjdb‐db/issues/193

**FIGURE 7:**
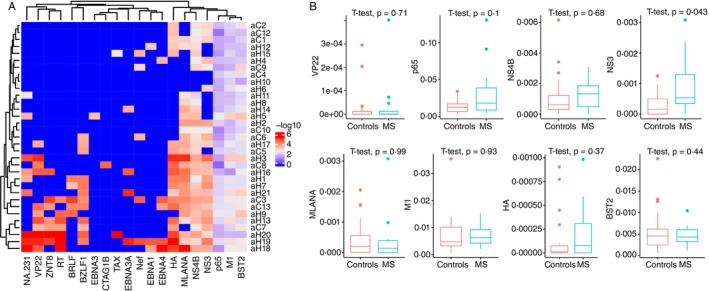
Antigen‐specific TCR‐α clonotype frequencies in MS and HC groups. (A) Heatmap of CDR3 TCR‐α clonotype annotations. (B) Comparative analysis of CDR3 TCR‐α clonotypes targeting viral and autoimmune‐related antigens, including VP22, p65, NS4B, MLANA, M1, HA and BST2. NS3‐HCV‐specific CDR3 clonotypes are significantly enriched in MS patient.

To further explore the potential link between infectious disease‐related and autoimmune‐related clonotypes, we annotated the CDR3 TCR‐α clonotypes from both MS patients and HCs. For most antigens—including VP22, p65, NS4B, MLANA, M1, HA and BST2—no significant differences in CDR3 clonotype frequencies were observed between the two groups (Figure 7). However, the NS3‐HCV‐specific CDR3 clonotypes were significantly increased in MS patients, suggesting a possible role for HCV‐specific TCR‐α repertoires in MS pathogenesis.

## 4 Discussion

This study is the first to employ deep sequencing technology to analyse TCR repertoires in Sardinian patients with MS. A major finding is the significant alteration of the TCR repertoire in MS patients, including changes in CDR3 length distribution, biased usage of V and J gene segments, and identification of MS‐specific clones, particularly those associated with MAIT and iNKT cells. These findings provide novel insights into the immune mechanisms underlying MS, further supporting the importance of TCR repertoires in autoimmune diseases and suggesting potential avenues for diagnostic biomarkers and targeted therapies.

Interestingly, we did not observe a significant shift in overall TCR diversity, which contrasts with some previous studies. For instance, studies by Muraro et al. [34] and Shugay et al. [24] reported variations in TCR diversity depending on disease stage or treatment context. In our study, while total diversity remained unchanged, MS patients exhibited higher TCR entropy compared to HCs. This suggests that while diversity may remain similar, the TCR repertoires in MS patients are more heterogeneous. This finding may reflect differences influenced by disease stage, therapies or other factors, and it could be influenced by the chronic phase of the disease in our patient cohort, contrasting with studies focused on acute or relapsing MS stages.

A particularly notable finding was the altered CDR3 length distribution, with longer CDR3 regions observed in MS patients. This shift suggests that MS patients may have a more diverse immune response, possibly indicating the presence of auto‐reactive T‐cell clones. Longer CDR3 regions are associated with broader antigen recognition [35], which supports the hypothesis of T‐cell dysregulation in MS, where expanded T‐cell clones may recognise self‐antigens and contribute to autoimmune processes in the CNS.

We also identified biased usage of certain TCR V and J gene segments, such as TRAV12.3, TRAV5, TRBV23.1 and TRBV3.1, which have been implicated in other autoimmune diseases [24]. These results reinforce the idea that certain TCR gene segments may play a role in the MS disease mechanism, possibly reflecting antigen‐specific T‐cell responses. The biased usage of these segments suggests that specific T‐cell subpopulations may target CNS self‐antigens, thereby participating in the autoimmune process. Our findings contribute new insights to the existing literature and encourage further exploration of the molecular mechanisms behind these TCR gene preferences.

An intriguing observation was the preferential usage of the TRBV20‐1 gene in MS patients (*p* = 0.0792), paired with specific J genes. This usage, more prominent in MS patients than in HCs, has not been previously reported in the Sardinian MS population. TRBV20‐1 has been linked to autoimmune diseases and viral infections, suggesting that its preferential use may be related to the pathogenesis of MS [24]. This finding aligns with studies by Shugay et al. [36] and Kotzen et al. [37], which highlighted the role of TCR‐β repertoires in MS immunity. Further research should explore TRBV20‐1's role in antigen recognition and its interaction with MHC molecules, as well as its contribution to disease progression. These findings also suggest that the use of longer V/J gene segments may influence CDR3 length, underscoring the need for further exploration of antigen recognition and disease mechanisms. The high frequency of TCR rearrangements in MS patients also suggests that these TCR clones may serve as potential biomarkers.

Additionally, we observed significant changes in the TCR‐α repertoire, particularly in relation to MAIT cells and other innate‐like T‐cell populations. T‐cell clones resembling those of MAIT cells (e.g., CAVLDSNYQLIW) were identified in MS patients, supporting the hypothesis of their involvement in the immune response. Though the role of MAIT cells in MS remains controversial, their presence in MS patients suggests they may contribute to both autoimmune responses and viral infections, such as cytomegalovirus (CMV) [38] and EBV. Notably, EBV reactivation has been reported in many MS patients, further emphasising the potential interplay between viral reactivation and immune responses in MS [39, 40].

A limitation of our study is the small sample size and focus on chronic‐phase MS patients, which may not fully reflect immune responses in earlier or relapsing disease stages. Future studies should involve longitudinal cohorts to track how TCR repertoires evolve throughout disease progression. Additionally, while we assessed TCR repertoires, we did not explore the antigen specificity of identified clones. Identifying specific antigens recognised by these TCRs, particularly within the CNS, is critical to understanding autoimmune mechanisms and identifying therapeutic targets. Given that MS treatments may influence TCR repertoires, it is also important to examine how therapies impact these changes. The application of advanced techniques, such as single‐cell sequencing and computational modelling, will be essential for elucidating the antigen specificity and functional roles of TCR clones, offering deeper insights into MS pathogenesis and providing potential therapeutic strategies.

## 5 Conclusion

In conclusion, our study reveals significant alterations in the TCR repertoire of MS patients, including longer CDR3 lengths, biased gene segment usage, and MS‐specific clonotypes. These findings provide valuable insights into the immune mechanisms underlying MS and suggest potential biomarkers and therapeutic targets. By comparing our results with previous studies, we have broadened our understanding of the TCR landscape in MS and its role in autoimmune responses. While the study is limited by factors such as small sample size and the absence of antigen specificity data, it lays the groundwork for future investigations into the molecular mechanisms of MS and the potential role of infections in triggering autoimmunity. Further research into the identified TCR clonotypes and their functional implications could pave the way for novel therapeutic strategies in MS management.

## Ethics Statement

The study was approved by the CE ASL1 prot 2150/CE.

## Conflicts of Interest

The authors declare no conflicts of interest.

## Data Availability

The data that support the findings of this study are openly available in ENA at https://www.ebi.ac.uk/ena/browser/view/PRJEB89256, reference number PRJEB89256.
